# Green Approaches to Sample Preparation Based on Extraction Techniques

**DOI:** 10.3390/molecules25071719

**Published:** 2020-04-09

**Authors:** Alshymaa A. Aly, Tadeusz Górecki

**Affiliations:** 1 Department of Chemistry, University of Waterloo, Waterloo, ON N2L 3G1, Canada; a3alimoh@uwaterloo.ca; 2 Analytical Chemistry Department, Faculty of Pharmacy, Minia University, Menia Governorate 61519, Egypt

**Keywords:** green analytical chemistry, ecofriendly extraction techniques, environmentally friendly analytical processes

## Abstract

Preparing a sample for analysis is a crucial step of many analytical procedures. The goal of sample preparation is to provide a representative, homogenous sample that is free of interferences and compatible with the intended analytical method. Green approaches to sample preparation require that the consumption of hazardous organic solvents and energy be minimized or even eliminated in the analytical process. While no sample preparation is clearly the most environmentally friendly approach, complete elimination of this step is not always practical. In such cases, the extraction techniques which use low amounts of solvents or no solvents are considered ideal alternatives. This paper presents an overview of green extraction procedures and sample preparation methodologies, briefly introduces their theoretical principles, and describes the recent developments in food, pharmaceutical, environmental and bioanalytical chemistry applications.

## 1. Introduction

In recent years, awareness of the impact of dangerous solvents on the environment has increased significantly. Many developments have been introduced to reduce this impact and protect the environment. If a development meets the requirements of the current generations without affecting the needs of future generations, it is considered sustainable [1]. The overarching goal of sustainable development is to enhance the quality of life, even at a cost of certain restrictions to human actions. Technologies that mainly involve chemical activities at both industrial and laboratory scale are considered major factors influencing sustainability. Even small-scale activities of chemists, such as laboratory experiments utilizing significant volumes of hazardous chemicals, have the potential to negatively affect the environment in case of uncontrolled disposal of chemical waste [2].

Various measures have been developed to mitigate the impact of chemical activities and to protect the environment and the chemists that are often in direct contact with hazardous chemical reagents and samples (environmental, biological, clinical, etc.) [3,4,5,6]. A variety of terms such as “green chemistry”, “environmentally benign chemistry”, “clean chemistry”, etc. [7], have been introduced to emphasize the need to perform chemical activities in an environmentally friendly way. These approaches rely on minimizing the consumption of reagents and the generation of waste, and eliminating hazardous chemicals [8]. The concept of “green chemistry” was introduced in 1998 by Anastas [9]. Green analytical chemistry (GAC), an offshoot of green chemistry, relies on green chemistry principles to increase the safety of operators, decrease energy consumption, properly manage wastes, minimize or even eliminate the use of hazardous chemicals and replace them with benign ones whenever practical [10,11].

In practice, four factors—sample collection, sample preparation, reagents, and instrumentation, are considered significant in greening analytical methods [2]. Preparing the sample prior to analysis is a fundamental part of many analytical protocols. As a result, it is very important to make this step greener, especially if samples are prepared from complex matrices. Recent progress in sample preparation techniques have been focused on automation, miniaturization and simplification of the extraction procedures [12].

Several books devoted to this area have been published, such as *Green Analytical Chemistry* [13], *Green Analytical Chemistry: Past, Present and Perspectives* [14], and *Handbook of Green Analytical Chemistry* [15]. In addition to this, several review articles presenting some of these green sample preparation techniques have been published in the last twenty years [1,14,16,17,18,19,20,21]. Herein, an overview of various benign sample preparation and extraction techniques, their theoretical principles and recent applications will be described in detail.

## 2. Ecofriendly Extraction and Sample Preparation Procedures

### 2.1. Direct Analysis without Sample Pretreatment

Various steps including drying, grinding, dissolution, etc. are necessary to treat solid samples before analysis. Additional steps such as distillation, evaporation, precipitation or re-crystallization might be required before measurement to increase the concentration of the target analyte(s) in the sample to match the sensitivity of the instrument. Numerous instruments require liquid samples, which have to be prepared using large volumes of organic solvents that have the potential to seriously affect both the environment and the chemists.

To limit the impact of solvents on the environment, the preferred approach is to use direct analysis, which eliminates the need for sample preparation. However, direct methodologies are usually appropriate only when analyzing clean matrices [22]. Nevertheless, they have been successfully implemented in both gas and liquid chromatography. Sample preparation-free gas chromatographic analysis based on direct on-column injection was introduced in 1978 [23]. Other applications adopted direct injection to analyze various compounds in water samples, such as halogenated compounds [24], volatile organic compounds [25], volatile organic components with various polarities [26] and high-boiling volatile organic compounds [27]. An example of direct analysis in liquid chromatography is the determination of pesticides in water samples without sample pretreatment [28]. Other analytical procedures requiring only simple sample pretreatment, such as filtration [29], dilution [30] or centrifugation [31], can be considered direct green techniques.

### 2.2. Green Sample Preparation Methodologies

Sample preparation is the most time-consuming step in many methodologies, especially those used for complicated matrices. In order to make this step more benign, various approaches including miniaturization, simplification and automation of extraction procedures have been adopted. Following is an overview of the most common green sample preparation methods focusing mostly on organic analytes.

#### 2.2.1. Solid Phase Extraction (SPE)

SPE is one of the most widely used sample preparation techniques. In SPE, an aqueous sample is passed through a short column containing a suitable solid sorbent, and the solutes are adsorbed onto the column. Afterwards, small amounts of organic solvents of high elution strength are used to recover the analytes from the sorbent, which leads to their enrichment [32]. Solid phase extraction utilizes small amounts of solvent and generates little waste. As a result, it is considered an ecofriendly technique. Despite the merits of this technique, it has some potential downsides that must be considered in order to avoid inefficient extraction of analytes. One of the issues is the non-uniformity of the packing material bed, which may lead to efficiency loss. The use of commercial cartridges is a safe way to alleviate this problem. In addition, limited selectivity of some conventional sorbents may lead to insufficient retention of very polar compounds [33]. Another issue is the competition between the analytes and the sample matrix for retention, which can dramatically impact analyte recovery. Consequently, careful optimization of the method is required to ensure effective extraction of analytes [34].

SPE has been implemented as a green sample preparation method in a large variety of applications. Several review articles presented selected applications of SPE in different areas [34,35,36,37,38,39]. Some examples of SPE applications are listed in Table 1.

##### QuEChERS Extraction Methodology

QuEChERS is a popular extraction method which is renowned for its quick, easy, cheap, effective, rugged and safe characteristics. The initial letter(s) of each of these words form the acronym under which the technique is known. The detailed QuEChERS procedure was introduced in 2002 by Anastassiades et al. [73]. This technique utilizes small volumes of organic solvents compared to other extraction procedures, and that is why it is considered a green extraction technique. QuEChERS procedures involve two main steps: solvent extraction and sample clean up. The solvent extraction step is performed via vigorous shaking of the sample with acetonitrile (extraction solvent), anhydrous magnesium sulfate and sodium chloride (salting out), and buffer (to protect base-sensitive analytes in the sample). The sample cleanup step is crucial in order to eliminate interfering matrix compounds such as fatty acids and carbohydrates. This is done through quick dispersive solid phase extraction to clean the extract using magnesium sulfate (to absorb residual water) and a weak anion exchanger called “primary secondary amine” (PSA) sorbent as dispersive solid-phase extraction agent [74].

Initially, QuEChERS was considered the method of choice in the analysis of pesticide residues in vegetables and fruits [75]. More recently, it has found numerous applications in the analysis of a plethora of other compounds in food and other types of matrices. Several reviews have presented the applications of QuEChERS as an effective extraction methodology [76,77,78]. Prominent examples of QuEChERS applications in food analysis include the analysis of mycotoxins in rice [79], quinolones in fish by high performance liquid chromatography (HPLC) [80], acrylamide in Sudanese food [81], determination of sulfonamide residues in pasteurized milk [82], as well as pesticide residues in honeybees [83] and fruits and vegetables [84,85]. In addition to this, some applications employed QuEChERS to extract analytes of interest from blood samples, including extraction of 40 pharmaceutical drugs from blood before determination by gas chromatography-mass spectrometry (GC-MS) [86], removal of various contaminants from human blood samples [87], extraction of amphetamines, opiates and cocaine metabolites from blood before analysis by liquid chromatography-tandem mass spectrometry [88], and recently complete extraction of tetrahydrocannabinol and its metabolites from blood samples followed by gas chromatography-tandem mass spectrometry [89]. 

##### Solid Phase Microextraction (SPME)

SPME was introduced by Belardi and Pawliszyn in 1989. According to the GAC guidelines, it is a green extraction procedure because it avoids the use of organic solvents and combines extraction, enrichment and sample injection into a single step. Analytes can be efficiently extracted by SPME from liquid or gas samples by absorption into or adsorption onto a thin layer of polymer attached to a solid surface of a fiber fixed inside a capillary or an injection needle. Analytes partition between the sample matrix and the SPME fiber coating when the fiber is immersed directly in the sample (direct immersion, or DI-SPME), or between the sample headspace and the fiber coating when the fiber is placed in the space above the sample (HS-SPME). Partitioning continues until equilibrium is established between all phases involved. When the extraction process is completed, the SPME fiber is transferred directly to the analytical instrument of choice, typically a gas chromatograph, where analyte desorption takes place. The major advantages of SPME include low cost, simplicity, elimination of the solvent disposal costs, short sample preparation time, reliability, sensitivity, and selectivity. Nevertheless, there are some drawbacks which should be pinpointed to avoid poor results. They include the fragility of the fiber, which may be easily broken during handling, the potential damage of the fiber coating if it is used repeatedly, competitive sorption when adsorbent-type coatings are used, pronounced effect of temperature and mass transfer conditions on equilibration, matrix effects, etc. Consequently, adequate quality control and quality assurance measures are essential when using SPME. 

SPME has been employed successfully numerous times as an ecofriendly sample preparation technique combined with GC or GC-MS prior to analysis of organic compounds in complicated sample matrices [90]. In addition to this, SPME has also been used effectively in combination with high-performance liquid chromatography (HPLC) in the analysis of thermally unstable or poorly volatile compounds which cannot be analyzed by gas chromatography [90]. However, SPME is not a completely solvent-free technique when combined with HPLC because small volumes of the organic solvent are necessary for desorption of the analytes from the fiber prior to HPLC separation. Numerous reviews have presented the applications of SPME in the analysis of various samples, e.g., [91,92,93,94,95,96,97,98,99,100,101,102,103,104]. Table 2 illustrates some representative examples of SPME applications.

##### Microextraction by Packed Sorbent (MEPS)

MEPS uses the same sorbents as SPE. However, in this technique, analytes are concentrated onto a small amount of a sorbent integrated into a syringe. The analytes are recovered from the sorbent using a small volume of a suitable solvent. The liquid extract is then injected into a chromatographic system for further analysis, and sorbent regeneration can be effectively achieved by washing it with additional portions of a solvent. MEPS is considered more advantageous than SPE because sorbent integration into a liquid-handling syringe results in low void volume and makes sample manipulations easy. Furthermore, MEPS procedures significantly reduce the solvent volume compared to conventional solid phase extraction (about 10 µL vs. several mL for the latter). As a result, the extraction time can be reduced to as little as one minute with minimal solvent and energy consumption. In addition to this, MEPS can be performed online in a fully automated fashion using the same syringe for sample extraction and extract injection into the analytical instrument. Numerous applications employing MEPS as an environmentally friendly extraction technique for various matrices have been described, including tricyclic antidepressant drugs in human oral fluid [137], non-steroidal anti-inflammatory drugs (NSAIDs) in urine and human plasma [138], brominated diphenyl ethers in sewage sludge [139], clenbuterol in pork samples [140], drugs of abuse in plasma (cocaine, amphetamines and opiates) [141] and human hair [142], various metabolites in urine [143] as well as risperidone and clozapine with their metabolites in urine [144].

##### Stir-Bar Sorptive Extraction (SBSE)

SBSE is a green, solvent-free extraction technique which was introduced in 1999 as an alternative to SPME. SBSE was initially used for the extraction and enrichment of volatile analytes from aqueous matrices. Its applications have been extended later to the analysis of the headspace over solid or liquid samples, gaseous samples, as well as nonvolatile analytes in combination with HPLC. Like SPME, SBSE involves sorptive extraction. However, in this case analytes are absorbed into a layer of a polymer (e.g., polydimethylsiloxane; PDMS) coated on a magnetic stir rod rather than a fiber. Extraction efficiency depends on the partitioning coefficient of the analytes between the sample and the coating. The major difference between SBSE and SPME is the much larger volume of the sorptive phase used with the former. This results in better sensitivity, especially for compounds with low partition coefficients or when using large sample volumes. Volatile analytes extracted by the coating can be thermally desorbed directly into a GC, making this technique in this case completely solvent-free. Nonvolatile analytes are desorbed with small volumes of a solvent, maintaining the green character of the method.

Examples of SBSE applications include determination of volatile compounds in cooked cured pork ham [145], volatile organic compounds in human urine, and water samples [146], organic pollutants in water samples [147], free fatty acids in the exudates of cooked ham [148], barbiturates in urine [149], coffee volatiles in roasted Arabica coffee [150], organic pollutants in water samples [151] and odorous compounds in drinking water [152]. Recently, SBSE has been used for enhanced recovery of pesticides in wines and aromatic compounds in beers [153] and selective enrichment and analysis of polychlorinated biphenyls in fish [154].

##### Solid Phase Nanoextraction (SPNE)

SPNE is based on strong affinity of analytes to nanoparticles. In this extraction technique, an aqueous sample is mixed with a colloidal solution of nanoparticles. Analytes bind to the nanoparticles’ surface in a very short time. Afterwards, centrifugation is performed to recover the nanoparticles, and the analytes are recovered from them using various solvents. There have been several applications which adopted SPNE as a green extraction technique, e.g., the analysis of PAHs (polycyclic aromatic hydrocarbons) in drinking water [155,156] and polychlorinated biphenyls in environmental waters [157].

#### 2.2.2. Liquid-Phase Microextraction Techniques (LPME)

LPME techniques use a small volume of an organic solvent (typically 1–100 μL) to extract the analytes. The major modes of LPME are single drop microextraction (SDME), hollow fiber liquid-phase microextraction (HF-LPME) and dispersive liquid-liquid microextraction (DLLME).

##### Single Drop Microextraction (SDME)

SDME, also referred to as liquid–liquid microextraction (LLME), involves the distribution of the analytes between an aqueous sample and a small droplet of a solvent suspended at the tip of a microsyringe needle. Once equilibrium between the two phases is reached, the microdrop is retracted into the syringe, and introduced to the analytical system. If the drop is directly immersed in the aqueous sample, the technique is called direct immersion SDME (DI-SDME); when the drop is fixed in the headspace above the sample, the technique is called headspace SDME (HS-SDME) [158]. Extraction efficiency in SDME is dependent on several parameters, including extraction time, the nature of the organic solvent and the analytes, stirring conditions, organic drop volume, etc. These parameters must be carefully optimized during method development to obtain the best results [159].

SDME has become popular because it is a nearly solvent-free extraction technique (only a few µL of a solvent are required). In addition to this, it is inexpensive, easy to operate, can be used to highly enrich analytes in a relatively short time, and can be conducted using very basic apparatus. These features make this technique more advantageous than other microextraction techniques in the eyes of many users. SDME has been widely employed as a green microextraction technique for trace analysis in chemical, biological, food, environmental, clinical, pharmaceutical, and forensic analysis. Representative examples include analysis of Pd, Be, Co, and Cd in biological samples [160], methamphetamine in human plasma and urine [161], carbaryl and triazophos pesticides in fruit juice and water samples [162], organic compounds in environmental matrices [163], xylene, toluene, benzene, and ethylbenzene in water samples [164], essential oils in plants and herbs [165], accelerants of fire in fire debris samples [166], volatile organic compounds in water and air [167], phenol derivatives in dairy products and soft drinks and organophosphorus pesticides in juice [168].

##### Hollow Fiber Liquid-Phase Microextraction (HF-LPME)

This microextraction technique utilizes a porous capillary made of an inert material as a carrier for the extraction solvent. It can be operated in two modes [158]:

- Two-phase hollow fiber LPME. In this technique, an organic solvent fills the pores and lumen of a semipermeable membrane, which is a porous hollow fiber made e.g. of polypropylene. The membrane is immersed in the sample and analytes partition through the pores of the membrane into the organic solvent inside.
- Three-phase hollow fiber LPME. In this technique, an immiscible solvent fills the pores of the hollow fiber, which separates the sample from another solvent inside the lumen, so that two equilibria for the analytes take place. The first one is between the aqueous sample and the organic solvent in the capillary wall pores, and the second one is between the solvent in the pores and the solvent in the lumen. In other words, the analytes cross the organic solvent embedded in the holes of the semipermeable membrane and are concentrated into a third phase inside the capillary’s lumen.

HF-LPME has been utilized as a green microextraction methodology for multiple analytes, such as chlorophenols in water samples [169], organic contaminants in the aquatic environment [170], pesticides and their metabolites in water and soils [171], antidepressants in vitreous humor [172], NSAIDs in sewage sludge [173], amino alcohols in water samples [174], anabolic steroids in urine [175], selective serotonin reuptake inhibitors in sewage sludge [176], basic drugs in water [177], haloacetic acids in water [178], cocaine and its derivatives in hair [179], bisphenol A in river water samples [180], organochlorine pesticides in water [181], pesticides in environmental water [182,183], chloroanilines in water samples [184], aromatic amines in environmental water samples [185], paraben preservatives in cosmetic samples [186], short-chain fatty acids in plasma [187], fungicides in water [188], food analysis [189,190], nortriptyline in biological matrices [191], aromatic hydrocarbon isomers [192] and pesticide residues in food samples [193].

##### Dispersive Liquid-Liquid Microextraction (DLLME)

DLLME is a novel addition to LPME. It was introduced in 2006 by Rezaee et al. [194]. The technique involves a three-phase system, which consists of an aqueous sample, a water-immiscible extracting solvent, and a dispersive solvent that is miscible with both phases. The two solvents are mixed together, and the mixture is injected rapidly into the sample forming a very fine emulsion. This allows very fast transfer of the analytes into the dispersed extraction solvent. The emulsion formed is then centrifuged, and the high-density extracting phase is collected with a microsyringe and introduced into the analytical instrument of choice. DLLME offers several advantages including small sample volume, low consumption of solvents, high enrichment factor, good repeatability, and high recovery. The fine emulsion formed after the addition of the solvent mixture results in a large contact area between the aqueous phase and the extracting solvent. Consequently, the equilibration is fast and the extraction is efficient.

All types of LPME are equilibrium techniques. They are particularly suitable for the extraction of nonpolar analytes from aqueous samples. The presence of the water-miscible dispersive solvent may lead to the reduction of the analyte’s partition coefficient between water and the extracting solvent. To mitigate this limitation, dispersion of the extraction solvent in the sample can be achieved by the application of ultrasounds [195], vortexing [196,197] or air [198]. When ultrasounds are used in order to disperse the extraction solvent in the sample, the technique is called US-DLLME.

DLLME has been applied for the extraction of numerous analytes from various matrices, including phthalate esters in soybean milk [199] and in commercial beverages [200], milk and dairy products [201], biogenic amines in meat [202], methylated polycyclic aromatic hydrocarbons in water [203], tetrabromobisphenol A from dust samples [204], organochlorine pesticide residues in honey [205], amines in home-made fermented alcoholic drinks [206], chlorinated phenols in water samples [207], volatile organohalogen compounds in drinking water [208] and aryloxyphenoxy-propionate herbicides in water [209]. Other applications which adopted US-DLLME included the analysis of endosulfan and its metabolites in urine and soil samples [210] and pyrethroids in soil [211].

Another development in DLLME was the utilization of ionic liquids as alternative extraction solvents rather than toxic chlorinated solvents. This technique has been named IL-DLLME. It is considered a green technique because it avoids the use of toxic solvents and can be performed without using a disperser solvent. When ionic liquids are adopted for extraction of aqueous samples, the system can be heated until it is homogeneous, then cooled to promote phase separation. Afterwards, the ionic liquid with the analytes is isolated by centrifugation. Procedures based on this principle are called temperature-controlled IL-DLLME. Numerous applications of IL-DLLME have been developed, including determination of pesticides and their metabolites in soils [212], copper in natural waters [213], chlorophenols in honey samples [214], organophosphorus pesticides in wheat [215], phenols [216], trihalomethanes in water [217], polycyclic aromatic hydrocarbons [218], and organophosphorus pesticides in environmental samples [219].

#### 2.2.3. Membrane Extraction

These procedures utilize nonporous membranes that can be a solid (polymer impregnated with a liquid) or a liquid, and which are fixed between two other phases, usually liquid, but sometimes gaseous.

##### Supported Liquid Membrane Extraction (SLME)

SLME is based on the same principle as three-phase HF-LPME; there is variation in solute concentration between donor and acceptor phases. Adjusting the concentration gradient between these two phases can be achieved by adjusting the pH; the solutes exist in nonionized form in the donor phase, and ionized form in the acceptor phase. Analytes in nonionized form in the donor phase can be easily extracted into the membrane, while the ionized form is irreversibly trapped in the acceptor phase. Afterwards, the acceptor phase is introduced into the analytical instrument of choice for the analysis. Examples of the use of SLME as an ecofriendly extraction technique include determination of metals in waste water [220], pesticides in water [221], phenols in water samples [222], black B dye in waste water [223] and basic drugs in human plasma [224].

##### Microporous Membrane Liquid–Liquid Extraction (MMLLE)

MMLLE technique is based on the same principle as two-phase HF-LPME, and the only difference is the name used for this technique. The analytes partition between the aqueous and organic phase across the membrane. MMLLE permits the extraction and enrichment of analytes which cannot be extracted by SLME [20]. MMLLE has been used for the extraction and enrichment of numerous compounds from various complicated matrices. Examples include determination of polycyclic aromatic hydrocarbon in soil [225], organotin compounds [226], sulfonylurea herbicides in water samples [227] and pesticide residues in red wines [228]. 

##### Membrane Extraction with a Sorbent Interface (MESI)

This technique was first described more than two decades ago. It is a solvent-free technique that allows direct introduction of preconcentrated analytes to a gas chromatograph. MESI can be used to monitor semivolatile and volatile organic compounds on site. It requires no sample preparation, and the potential for analyte losses is dramatically reduced. MESI preconcentrates analytes during the extraction process, enhancing the sensitivity of trace analysis.

MESI is based on partitioning of the analytes from a gas or a liquid phase into a nonporous polymeric membrane. The analytes permeating through the membrane are then released from the inner membrane surface into a stream of gas passing through the extraction module. The gas carries the analytes to a suitable sorbent placed in-line, which traps and concentrates them. The enriched analytes are periodically thermally desorbed and directly introduced into a GC column by a stream of carrier gas. MESI is thus a solvent-free membrane extraction technique which can be applied to on-site analysis and produces semicontinuous data, meeting numerous requirements of GAC. It has been applied for the extraction of various analytes, including biogenic emissions from eucalyptus leaves [229], toluene, benzene, xylene and ethylbenzene in water [230], volatile organic compounds in breath [231] as well as aromatic hydrocarbons in soil, water and air [232]. MESI was also applied for field analysis [233], analysis of human breath [234,235] and characterization of ethylene in human breath [236].

##### Membrane Assisted Solvent Extraction (MASE)

MASE is a substitute for standard liquid–liquid extraction (LLE). It is a small-scale LLE method utilizing a low-density polyethylene (LDPE) membrane, which isolates the aqueous sample from the organic solvent. Organic compounds in the aqueous sample are transferred across the polymeric membrane to a small amount of organic solvent, which is immiscible with water and is able to dissolve them. The extraction is typically performed at elevated temperature to accelerate mass transfer of the analytes into the solvent. The extract is then analyzed by gas chromatography. Since MASE uses only small amounts of organic solvents for the extraction, it is considered a green technique. MASE applications include the analysis of synthetic musks in water [237], endocrine disrupting compounds in wastewater [238], organophosphorus pesticides in water [239], phenols [240], tetramines in food [241], organophosphorus compounds in complex samples [242] and polycyclic aromatic hydrocarbons in wastewater [243].

##### Green Agarose Gel Electro-Membrane Extraction 

Pedersen-Bjergaard introduced electro-membrane extraction (EME) in 2006 [244]. EME involves electric field-driven movement of charged cationic and anionic analytes from a donor into an acceptor phase via a hollow fiber membrane inserted between the two phases. The hollow fiber membrane is saturated with an organic solvent before extraction [245]. To avoid the use of organic solvents, agarose gel membrane was introduced as a green solvent-free alternative for EME [246]. This membrane offers several advantages compared to conventional EME membranes, such as easy preparation, wide availability, eco-friendliness and ability to extract polar compounds. Agarose gel membrane was adopted for the extraction of verapamil, rivastigmine, amlodipine and morphine. It was also coupled with dispersive liquid–liquid microextraction to extract basic drugs from biological fluids [247]. 

##### Microdialysis Sampling

Microdialysis is a minimally invasive sampling technique that can be used to enrich hydrophilic analytes with low molecular weight in vivo. This technique is based on placing a probe with a semipermeable membrane in the sample. An electrolyte solution is then pumped through the probe, creating a concentration gradient between the perfusate and the surrounding medium. The analytes are transferred into the perfusate because of this concentration gradient. The dialysate containing the enriched analytes is then injected into an analytical instrument. Microdialysis sampling is more advantageous than conventional LLE because it requires small samples and solvent volumes. There have been numerous applications for microdialysis in pharmacokinetic and drug metabolism studies to monitor biologically active compounds in vivo [248,249,250,251,252]. In addition to this, it has been widely employed for extracting some analytes such as sugar in milk products [253], pesticide residues in a jade plant [254], metal ions [255], alachlor and its metabolites in microbial culture medium [256], aniline and 2-chloroaniline in industrial wastewater [257] and recently proteins, cytokines and metabolites in wounds [258].

##### Thin-Film Microextraction (TFME)

The technique is based on the same principle as SPME, except that it uses a thin film of the extraction phase rather than a polymer coating on a fiber [259]. It is an ecofriendly solventless microextraction methodology which was used for the extraction of PAHs from water [260].

#### 2.2.4. Green Alternatives to Extraction Solvents

##### Subcritical Water Extraction (SWE)

SWE is considered one of the greenest methodologies because water is naturally abundant, nontoxic, non-flammable, non-corrosive, environmentally safe and available at low cost. Nevertheless, water has some drawbacks that limit its use as a universal extraction solvent, such as low solubilizing power for nonpolar compounds, and high energy consumption during the extraction process. SWE is based on using superheated water instead of an organic solvent in the extraction process; below the critical point (P_c_ = 218 atm, T_c_ = 374 °C) water can extract polar analytes at low temperatures, whereas moderately polar or nonpolar organic analytes require higher temperatures for effective extraction. SWE is also called pressurized water extraction (PWE) or hot water extraction (HWE). SWE has been successfully used for the extraction of fatty acids, mannitol, antioxidants (phenols and flavonoids), sugars, resorcinol, essential oils, carotenoids, and pectin [261], polychlorinated biphenyls (PCBs) in soil [262], dioxins in soil [263], explosives and heavy metals in soil [264], anthocyanins from fruit berry [265], europium and yttrium from waste cathode-ray tube phosphor [266], oil and tea saponins [267], isoflavones from herbal plants [268] and pesticides in soil [269].

##### Supercritical Fluid Extraction (SFE)

In this technique, analytes are extracted from the matrix using supercritical fluids as the extracting solvents. SFE is primarily used for the extraction of organic compounds from solid matrices, but can be used for liquids as well. The process can be carried out in two modes: static and dynamic. In the static mode, the solvent is added to the sample and the mixture is left for a certain time at the required temperature and pressure. The dynamic mode involves the supercritical fluid continuously flowing through the sample. In both modes, the fluid needs to be depressurized to release the analytes, which are then collected in a solvent or using a solid sorbent, or are transferred directly to a chromatographic system. The most popular solvent used in SFE is carbon dioxide because it is non-corrosive, non-explosive, easily available and inexpensive. SFE minimizes or completely eliminates the use of hazardous organic solvents, earning it green credentials. It has been used for the extraction of petroleum hydrocarbons in soil [270,271,272], polychlorinated biphenyls (PCBs) in human adipose tissue [273], active constituents in medical plants [274,275], petroleum hydrocarbons (PHCs) in sand [276] and hazardous substances from solid and liquid matrices [277] among many others. Nowadays, SFE has been largely supplanted by pressurized liquid extraction in the analytical laboratories, and is used mainly for preparative purposes.

##### Ionic Liquids (ILs)

ILs are organic salts consisting of an organic or inorganic anion and a large organic cation, with melting temperatures below 100 °C. They offer unique characteristics such as non-volatility, negligible flammability, thermal stability (about ∼300 °C), strong solvation power for a wide variety of compounds and high ionic conductivity [278]. Consequently, they have been used as substituents for conventional organic solvents. They are sometimes regarded as ecofriendly solvents because they do not emit poisonous vapors to the surroundings. On the other hand, they can be toxic and form hazardous waste, thus the “green” designation is somewhat controversial. Research involving ILs is gradually moving toward the use of more biodegradable and less toxic formulations [279].

Most studied ILs consist of pyridinium, pyrrolidinium, imidazolium, phosphonium and tetraalkylammonium-based cations, attached to anions including bromide, acetate, chloride, bis(trifluoromethylsulfonyl)imide, tetrafluoroborate and hexafluorophosphate [280]. Ionic liquids have been utilized in extraction procedures including supported liquid membrane extraction [281], liquid–liquid microextraction [282] and dispersive liquid–liquid microextraction (DLLME) [283,284]. Table 3 shows some examples of extraction techniques that involved the use of ionic liquids as a benign alternative to conventional organic solvents.

##### Deep Eutectic Solvents (DES)

Abbott et al. introduced deep eutectic solvents for the first time in 2001 [299]. They are a promising alternative to the conventionally used volatile organic solvents. In addition to this, they have been deemed the most sustainable green replacement for ionic liquids, as they are manufactured from natural compounds such as organic acids, amino acids, sugars, alcohols, and cholinium derivatives. DES are prepared by combining a hydrogen bond donor (HBD) and a hydrogen bond acceptor (HBA). The strong hydrogen bond interactions between HBD and HBA result in a dramatic drop in the melting temperature of the mixture.

DES offer multiple advantages over ILs, including wide availability of their cheap natural individual components and easy preparation (no need for chemical synthesis). In addition to this, they are characterized by biodegradability, high solubilization power, adjustable polarity, wide range of liquid state and negligible volatility. However, they are less chemically inert than ionic liquids and highly viscous. As a result, the rate of mass transfer in dissolution or extraction processes is reduced. This drawback, caused by stronger Van der Waals bonds, hydrogen-bonding and electrostatic interactions between mixture components, can be mitigated by the addition of water during the preparation or as a diluent. DES have been used as green solvents for the extraction of various solutes, allowing for environmental monitoring of numerous pollutants in real matrices. Some representative examples of their applications are illustrated in Table 4.

##### Surfactants and Hydrotropes

Surfactants are amphiphilic chemicals which contain both hydrophobic and hydrophilic fragments in the molecule. Consequently, they are soluble in both aqueous and organic phases. In water, when the concentration of surfactant molecules exceeds the critical micellar concentration (CMC), the molecules arrange in micelle forms, in which the nonpolar chains are in the center of the micelle, whereas the polar heads are in contact with water. On the other hand, in nonpolar media, surfactant molecules arrange with the polar heads in the center of the structure to form reverse micelles [322].

Surfactants can be adsorbed onto inorganic surfaces forming a monolayer or a bilayer. In the monolayer assembly, the head groups of an ionic surfactant are attracted to the oppositely charged mineral oxide surface (silica, titanium oxide or alumina) resulting in a hemimicelle. In the bilayer or admicelle aggregation, a second layer is formed on top of the monolayer through the interaction between the nonpolar chains of the monolayer adsorbed to the surface and the surfactant molecules present in the solution. In this arrangement, polar heads of the surfactant molecules are in contact with water, while the nonpolar chains below form an organic core into which organic molecules can be dissolved [323]. The arrangement of hemimicelles or admicelles is dependent on pH, surfactant concentration and the ionic strength of the solution [324]. Surfactants adsorbed onto solid surfaces have been used for the extraction of multiple compounds. Table 5 illustrates some recent applications.

Hydrotropes, which are amphiphilic green solvents, solubilize hydrophobic compounds in the aqueous solutions by means other than micellar solubilization. They typically consist of a benzene-substituted molecule (hydrophobic part) and an ionic group (hydrophilic head). They are similar to surfactants; however, the hydrophobic part is too small to form a micelle through spontaneous self-aggregation [325]. At a particular concentration, which is known as the minimum hydrotrope concentration (MHC), hydrotropes can aggregate. This increases the solubility of hydrophobic compounds in the aqueous phase. Hydrotropes are nontoxic, inexpensive and chemically inert, and, as such, have found applications in the extraction of certain compounds [326] 

##### Bioderived (Agro) Solvents

Bioderived solvents are renewable materials developed to replace dipolar aprotic solvents. They are characterized by low toxicity, renewability, biodegradability and non-flammability. Examples of these biobased solvents include ethyl lactate, 2-methyl tetrahydrofuran, glycerol, ethanol, terpenes and p-cymene. Some of these solvents have been applied for the extraction of various analytes from different matrices. For example, d-limonene from citrus fruit [345,346] can be used for effective extraction of fats and oils [347]. Limonene, first described as a solvent in 2008 by Virot et al. [348], can be used as a replacement for petroleum-based solvents such as n-hexane and toluene in the extraction of some natural compounds [349]. Another example is the extraction of thymol, which is the main monoterpene phenol in thyme essential oil, by using limonene, ethanol and ethyl lactate [350]. Heated water/glycerol mixture was used to extract polyphenols from olive leaves, and the polyphenols yield was comparable to that obtained with ethanol/water mixture [351].

## 3. Conclusions

Determination of trace analytes in different matrices typically involves various sample preparation steps leading to isolation and concentration of the analytes prior to their final determination. The introduction of novel procedures aiming at the simplification, automation, and minimization of wastes of analytical methods has become inevitable to protect the environment and the analysts. Multiple environmentally friendly sample preparation methodologies have been developed over time, including solid phase extraction techniques, liquid phase microextraction techniques, and using alternative green solvents, such as subcritical water, ionic liquids, supercritical liquids, deep eutectic solvents, surfactants and hydrotropes, and bioderived solvents. As the attention paid to GAC principles is continuously growing, it is expected that the development of novel environmentally friendly extraction techniques will continue in the future.

## Figures and Tables

**Table 1:** Examples of solid phase extraction (SPE) applications in the analysis of various samples.

Analytes	Matrix	Technique	Ref.
Diethylstilbestrol, dienestrol and hexestrol	Urine and plasma	GC-MS	[40]
Salbutamol and clenbuterol	Liver, kidney, muscle	ELISA	[41]
Cd, Ni, Pb	Water	FAAS	[42]
Microcystins	Water	LC-MS	[43]
Metal dithiocarbamates	Water and tissues	FAAS	[44]
Imazalil	Citrus fruit	LC-MS	[45]
Glycans	Glycoproteins	MALDI-MS	[46]
Pyrethroid Bioallethrin	Fruit, vegetables, soil and dust	ELISA	[47]
Aflatoxins B1 and B2	Pistachio	IMS	[48]
Paralytic shellfish poisoning toxins	Shellfish	LC-MS	[49]
Fenoterol and fenoterol deravatives	Rat plasma	LC-MS	[50]
Chlorophenols	Water	GC-MS	[51]
Cyanazine	Water	TOF-SIMS, DRIFT	[52]
Pb, Cd, Ni, Zn, Fe, Cu, Co	Water	FAAS	[53]
Cationic surfactants	Surface water	Ion chromatography	[54]
Pyraclostrobin	Fruit juices	LC-UV	[55]
Chlorotriazine residues and dealkylated metabolites	Soil and water	LC-DAD	[56]
Cyclophosphamide	Surface water	HPLC-MS	[57]
Platinum	Water	ICP-MS	[58]
Hexapeptides	Antiwrinkle cosmetics	LC-MS	[59]
Polycyclic aromatic hydrocarbons	River, tap and mineral water	GC-MS	[60]
Cyclic guanosine and cyclic adenosine monophosphate	Human plasma and animal tissue	LC-MS	[61]
Nitrophenols	River water	CE-UV	[62]
Endogenous cytokinins	Plant	LC-MS	[63]
Proteins and phospholipids	Plasma	LC-MS	[64]
Bufadienolide	Traditional Chinese medicines	LC-MS	[65]
Indolic compounds	Plant extracts	HPLC-UV	[66]
Pyraclostrobin, picoxystrobin, and azoxystrobin	Water samples strawberry juice	IAC-IMS	[67]
Phosphatidylcholine and phosphatidylethanolamine	Salmon fish	MALDI-TOF	[68]
Zanamivir	Water	LC-MS	[69]
Aminoglycoside antibiotics	Honey	LC-MS	[70]
Carbamate pesticide residues	Fruit juice	UHPLC-MS	[71]
Tartrazine	Milk	LC-UV	[72]

GC: gas chromatography; MS: mass spectrometry; ELISA: enzyme-linked immunosorbent assay; FAAS: flame atomic absorption spectrometry; LC: liquid chromatography; MALDI: matrix-assisted laser desorption and ionization; IMS: ion mobility spectrometry; ToF-SIMS: time-of-flight secondary ion mass spectrometry; DRIFT: diffuse reflectance Fourier transform infrared spectroscopy; ICP-MS: inductively coupled plasma mass spectrometry; CE: capillary electrophoresis; UV: ultraviolet detector; IAC: immunoaffinity chromatography; UHPLC-MS: ultra-high performance liquid chromatography/mass spectrometry.

**Table 2:** Examples of solid phase microextraction SPME applications in the analysis of various samples.

Analytes	Matrix	Method	Ref.
Flavor volatiles	Garlic	HS-SPME-GC×GC-FID	[105]
Volatiles	Biological fluids	HS-SPME-GC/MS	[106]
Solvents	Pharmaceutical products	SPME-GC-FID	[107]
Fatty acids	Lung tissues	HS-SPME-GC/MS	[108]
Solvent residuals	Pharmaceutical samples	HS-SPME-GC	[109]
Polar volatile solvent residuals	Pharmaceutical samples	HS-SPME-GC/MS	[110]
Ethylbenzene, toluene, and xylene isomers.	Water, gas, and soil	HS-SPME-GC/MS	[111]
Dichloroanisoles, trichloroanisoles and pentachloroanisole	Cork	HS-SPME-GC-TOF-MS	[112]
Aroma compounds	Apricots	SPME-GC-MS	[113]
Flavor volatile compounds	Uncooked and cooked beef shanks, flanks, and ribs	HS-SPME-GC-MS	[114]
Volatile organic compounds	Water	HS-SPME-GC-MS	[115]
Nitrosamines	Latex products	HS-SPME-GC-MS	[116]
Volatile organic compounds	Lung cell lines	HS-SPME-GC-MS	[117]
Aldehydes	Human urine	HPLC	[118]
Manuka honey	A solid food model system	HS-SPME-GC/MS	[119]
Volatile compounds	Jelly bush honey	MAE–HS-SPME-GC-MS	[120]
Aroma volatile constituents	Vegetation materials	HS-SPME-GC/MS	[121,122,123,124]
Volatile contaminants	Fruits	HS-SPME-GC/MS	[125]
Nerolidol	Fruits	HS-SPME-GC	[126]
Pesticides	Tea	SPME-GC-MIP-AED	[127]
Pesticides	Herbal and tea infusions.	SPME-GC-MIP-AED	[128]
Manganese compounds	Honey	SPME-GC-MIP-AED	[129]
Butyltin compounds	Seawaters and soils	SPME-GC-MIP-AED	[130]
Volatile compounds	Aromatic rice grains	HS-SPME-GCxGC-MS	[131]
Biogenic amines	Wine	GC-MS	[132]
Lipids	Human breast milk	LC-MS	[133]
Polycyclic aromatic hydrocarbons	Airport runoff water	GCxGC-MS	[134]
Antifreeze substances	Airport runoff waters	GC-MS	[135]
Polar organic compounds	Water	GC-FID	[136]

HS-SPME: headspace solid phase microextraction; GC×GC: comprehensive two-dimensional gas chromatography; GC-FID: gas chromatography with flame ionization detection; GC-ToF-MS: gas chromatography/time-of-flight mass spectrometry; GC-MS: gas chromatography/mass spectrometry; GC-MIP-AED: gas chromatography/microwave-induced plasma atomic emission detection; HPLC: high performance liquid chromatography.

**Table 3:** Examples of applications of ionic liquids (ILs) for sample preparation.

Analytes	Matrix	Method	Ref.
Sulfonylurea herbicides	Wine samples	HPLC	[285]
Organophosphorus pesticides	Water	GC-MS	[286]
Polycyclic aromatic hydrocarbons	Water	HPLC	[287]
Zinc	Water and milk	FAAS	[288]
Bisphenol A	Human fluids	HPLC-MS	[289]
Emerging contaminants	Water	HPLC-UV/Vis	[290]
Fluoroquinolones and NSAIDs	Water	HPLC-DAAD	[291]
Aromatic amines	Water	HPLC	[292]
Organophosphate esters	water	GC-MS	[293]
Emerging pollutants	Water	HPLC-UV	[294]
Pollutants	Water	HPLC-UV	[295]
Triazine herbicides	Water	HPLC-UV	[296]
Neonicotinoids	Honey	HPLC-DAAD	[297]
Organophosphorus pesticides and aromatic compounds	Tap, rain and river water	HPLC-UV	[16]
Cadmium	Water	ETAAS	[298]

HPLC: high-performance liquid chromatography; FAAS: flame atomic absorption spectrometry; GC-MS: gas chromatography mass spectrometry; NSAIDs: nonsteroidal anti-inflammatory drugs; ETAAS: electrothermal atomic absorption spectrometry.

**Table 4:** Selected applications of deep eutectic solvents (DES) as green solvents in sample preparation.

Analytes	Matrix	Method	Ref.
Diazinon, metalaxyl, bromopropylate, oxadiazon, and fenazaquin pesticides	Fruit juice and vegetable samples	GC-FID	[300]
PAHs (phenanthrene, anthracene, fluoranthene and pyrene)	Water	GC-MS	[301]
PAHs (naphthalene, biphenyl, fluorine, acenaphthylene, fluoranthene and anthracene)	Marine biological samples (fish and microalgae)	HPLC-FL	[302]
Heavy metals (Zn, Fe, and Cu)	Fish	FAAS	[303]
Heavy metals (Cd, Pb, and Hg)	Soil and vegetables	FAAS	[304]
Ketoprofen, flurbiprofen and diclofenac	Lake water	HPLC-UV	[305]
Aromatic amines	Tap, surface and river water; wastewater	GC-MS	[306]
Anthocyanins	Grapes	HPLC-MS	[307]
Benzoylureas residual	River water, well water, and swimming pool water	HPLC-UV	[308]
Pesticides (imidacloprid, acetamiprid, nitenpyram, and thiamethoxam)	Water	UV-Vis	[309]
Flavonoids, terpene trilactones, procyanidine, polyprenyl acetates	Ginkgo biloba leaves	HPLC-UV	[310]
Lead and cadmium	Lipsticks and eye shadows	FAAS	[311]
Propionic acid, acetic acid, and butyric acid	Water	HPLC-UV	[312]
Caffeine, tryptophan, isophthalic acid and vanillin	Water	UV-Vis	[313]
Pesticides	Fruit juice	GC-FID	[314]
Amphetamine and methamphetamine	Human plasma, pharmaceutical waste water	HPLC-UV	[315]
Synthetic pigments	Tea beverages, carbonated drinks, fruit juices, lactobacillus beverages	HPLC-PDA	[316]
Malondialdehyde and formaldehyde	Human urine, apple juice, and rain water	HPLC-UV	[317]
Nitroaromatic compounds	Water	HPLC-UV	[318]
Polycyclic aromatic hydrocarbons	Industrial effluents	GC-MS	[301]
Caffeine	Green tea, cola and energy drink	HPLC-UV	[319]
Methylene blue	Wastewater and river water	UV-Vis	[320]
Pyrethroid pesticides	Tea beverages and fruit juices	HPLC-UV	[321]

GC–FID: gas chromatography with flame ionization detection; GC-MS: gas chromatography with mass spectrometric detection; FL: fluorescence detector; FAAS: flame atomic absorption spectrometry; UV: ultraviolet; Vis: visible; HPLC-PDA: high performance liquid chromatography equipped with photodiode array detector.

**Table 5:** Examples of applications of surfactants for solid phase extraction of multiple analytes from various matrices.

Analytes	Matrix	Method	Ref.
Three alkaloids	Oral liquid	HPLC-UV	[327]
PFASs and alkylphenols	Environmental water	HPLC-MS	[328]
Illegal cationic dyes	Chili sauce, soybean paste and tomato sauce	HPLC-DAAD	[329]
Alkyltrimethylammonium salts	Environmental water	HPLC-UV	[328]
Organophosphorus pesticides	Environmental water	HPLC-UV	[330]
Ibuprofen	Environmental water	HPLC-UV	[331]
Polycyclic aromatic hydrocarbons	Environmental water	HPLC-UV	[332]
Six fluoroquinolones	Environmental water	HPLC-UV	[333]
Benzodiazepines	Hair and waste water	HPLC-DAAD	[334]
2-Chlorophenol	Soil	UV-Vis	[335]
Sudan dye	Chilli sauce and ketchup	HPLC-UV	[336]
Lead	Water	FAAS	[337]
Heavy metals	Water	FAAS	[338]
Alkylphenols	Fruit juices	HPLC-MS	[339]
Perfluorinated carboxylic acids	Water	HPLC-MS	[340]
Acidic and basic pollutants	Water	HPLC-DAAD	[341]
Bisphenol A	Water	HPLC-UV	[342]
Sulfonamides	Environmental water	HPLC-UV	[343]
Heavy metals	Blood, amalgam and natural water	FAAS	[344]

HPLC-MS: high performance liquid chromatography with mass spectrometric detection; PFASs: perfluoroalkyl and polyfluoroalkyl substances; FAAS: flame atomic absorption spectrometry; UV: ultraviolet; Vis: visible.
